# Phenotypic Expression and Outcomes in Individuals With Rare Genetic Variants of Hypertrophic Cardiomyopathy

**DOI:** 10.1016/j.jacc.2021.07.017

**Published:** 2021-09-14

**Authors:** Antonio de Marvao, Kathryn A. McGurk, Sean L. Zheng, Marjola Thanaj, Wenjia Bai, Jinming Duan, Carlo Biffi, Francesco Mazzarotto, Ben Statton, Timothy J.W. Dawes, Nicolò Savioli, Brian P. Halliday, Xiao Xu, Rachel J. Buchan, A. John Baksi, Marina Quinlan, Paweł Tokarczuk, Upasana Tayal, Catherine Francis, Nicola Whiffin, Pantazis I. Theotokis, Xiaolei Zhang, Mikyung Jang, Alaine Berry, Antonis Pantazis, Paul J.R. Barton, Daniel Rueckert, Sanjay K. Prasad, Roddy Walsh, Carolyn Y. Ho, Stuart A. Cook, James S. Ware, Declan P. O’Regan

**Affiliations:** a MRC London Institute of Medical Sciences, Imperial College London, Hammersmith Hospital Campus, London, United Kingdom; b National Heart and Lung Institute, Imperial College London, London, United Kingdom; c Cardiovascular Research Centre at Royal Brompton and Harefield Hospitals, London, United Kingdom; d Biomedical Image Analysis Group, Department of Computing, Imperial College London, London, United Kingdom; e Department of Brain Sciences, Imperial College London, London, United Kingdom; f School of Computer Science, University of Birmingham, Birmingham, United Kingdom; g Department of Experimental and Clinical Medicine, University of Florence, Florence, Italy; h Cardiomyopathy Unit, Careggi University Hospital, Florence, Italy; i Wellcome Centre for Human Genetics, University of Oxford, Oxford, United Kingdom; j Faculty of Informatics and Medicine, Klinikum Rechts der Isar, TU Munich, Munich, Germany; k Department of Experimental Cardiology, Amsterdam UMC, AMC Heart Centre, Amsterdam, the Netherlands; l Cardiovascular Division, Brigham and Women’s Hospital, Boston, Massachusetts, USA; m National Heart Centre Singapore, Singapore; n Duke-NUS Graduate Medical School, Singapore

**Keywords:** cardiovascular magnetic resonance, deep learning, genetics, hypertrophic cardiomyopathy, penetrance, ACMG, American College of Medical Genetics and Genomics, CMR, cardiac magnetic resonance imaging, DCM, dilated cardiomyopathy, HCM, hypertrophic cardiomyopathy, LVH, left ventricular hypertrophy, MACE, major adverse cardiovascular events, P/LP, pathogenic or likely pathogenic, SARC-HCM-P/LP, pathogenic or likely pathogenic variants for hypertrophic cardiomyopathy in sarcomere-encoding genes, SARC-IND, indeterminate variants in hypertrophic cardiomyopathy–associated sarcomere-encoding genes (rare variants that do not meet criteria for pathogenic/likely pathogenic annotation), SARC-NEG, genotype negative/no rare variants in sarcomere-encoding genes, UKBB, UK Biobank, WES, whole exome sequencing, WT, wall thickness

## Abstract

**Background:**

Hypertrophic cardiomyopathy (HCM) is caused by rare variants in sarcomere-encoding genes, but little is known about the clinical significance of these variants in the general population.

**Objectives:**

The goal of this study was to compare lifetime outcomes and cardiovascular phenotypes according to the presence of rare variants in sarcomere-encoding genes among middle-aged adults.

**Methods:**

This study analyzed whole exome sequencing and cardiac magnetic resonance imaging in UK Biobank participants stratified according to sarcomere-encoding variant status.

**Results:**

The prevalence of rare variants (allele frequency <0.00004) in HCM-associated sarcomere-encoding genes in 200,584 participants was 2.9% (n = 5,712; 1 in 35), and the prevalence of variants pathogenic or likely pathogenic for HCM (SARC-HCM-P/LP) was 0.25% (n = 493; 1 in 407). SARC-HCM-P/LP variants were associated with an increased risk of death or major adverse cardiac events compared with controls (hazard ratio: 1.69; 95% confidence interval [CI]: 1.38-2.07; *P* < 0.001), mainly due to heart failure endpoints (hazard ratio: 4.23; 95% CI: 3.07-5.83; *P* < 0.001). In 21,322 participants with both cardiac magnetic resonance imaging and whole exome sequencing, SARC-HCM-P/LP variants were associated with an asymmetric increase in left ventricular maximum wall thickness (10.9 ± 2.7 mm vs 9.4 ± 1.6 mm; *P* < 0.001), but hypertrophy (≥13 mm) was only present in 18.4% (n = 9 of 49; 95% CI: 9%-32%). SARC-HCM-P/LP variants were still associated with heart failure after adjustment for wall thickness (hazard ratio: 6.74; 95% CI: 2.43-18.7; *P* < 0.001).

**Conclusions:**

In this population of middle-aged adults, SARC-HCM-P/LP variants have low aggregate penetrance for overt HCM but are associated with an increased risk of adverse cardiovascular outcomes and an attenuated cardiomyopathic phenotype. Although absolute event rates are low, identification of these variants may enhance risk stratification beyond familial disease.

Hypertrophic cardiomyopathy (HCM) is characterized by clinical and genetic heterogeneity, incomplete and age-dependent penetrance, and variable expressivity (1). Most individuals with HCM have a normal life expectancy but are at increased risk of adverse outcomes such as heart failure, atrial fibrillation, stroke, or sudden cardiac death (2).

A recent expert-led assessment of the validity of reported gene associations with HCM identified 8 sarcomeric genes with definitive evidence for disease causation (3), including *MYH7* and *MYBPC3,* that account for the majority of genetically explained disease (4). The American College of Medical Genetics and Genomics (ACMG) includes this set of genes among those for which specific variants are known to be causative of disease phenotypes and are clinically actionable (5). The ACMG recommends that these genes be analyzed whenever clinical exome sequencing is undertaken and that pathogenic or likely pathogenic (P/LP) variants should be proactively reported to patients as secondary findings. With increasing availability of whole exome sequencing (WES), both in wider clinical settings and as direct-to-consumer asymptomatic testing, this raises questions regarding potential benefit as well as downstream risks (6). Evidence is not currently available that would allow a critical evaluation of genomic screening at the population level (7). Specifically, it is unclear what risk cardiomyopathy-associated variants confer in the general adult population and their phenotypic expression outside families with penetrant disease. Current evidence is based on aggregating data from small and often underpowered studies (8), using different variant classifications and relying on inconsistent phenotyping.

Here, we sought to determine the population prevalence of rare sarcomeric variants in a prospectively recruited cohort of >200,000 middle-age participants drawn from the UK Biobank (UKBB) and to assess lifetime risk of adverse events. We analyzed 2 groups of variants found in 8 genes with definitive evidence for HCM (3): sarcomeric variants P/LP specifically for HCM (SARC-HCM-P/LP) and rare sarcomeric variants of indeterminate significance (SARC-IND) with the potential to cause HCM, dilated cardiomyopathy (DCM), or have little impact on cardiomyopathy risk. Using high-precision, deep learning phenotyping of cardiac magnetic resonance imaging (CMR), we also characterized phenotypic manifestations and estimated the prevalence of penetrant disease. Lastly, we determined the prevalence and genetic yield of sequencing in unexplained left ventricular hypertrophy (LVH) among this adult population.

## Methods

### Study cohorts

The UKBB recruited 500,000 participants aged 40 to 69 years across the United Kingdom between 2006 and 2010 (National Research Ethics Service, 11/NW/0382) (9). This study was conducted under terms of access approval number 40616. In each case, written informed consent was provided. The results are reported in accordance with the Strengthening the Reporting of Observational Studies in Epidemiology guidelines (10) and the checklist provided in Supplemental Table 1.

### Cardiac phenotyping using machine learning

**Figure 1:**
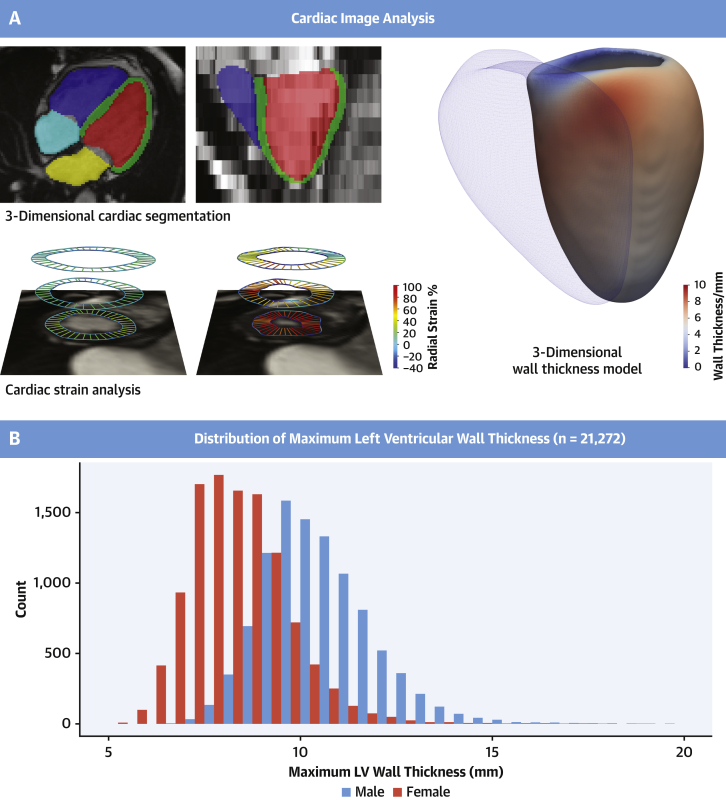
Cardiac Image Analysis in the UK Biobank **(A)** Machine learning segmentation of the heart from cardiac magnetic resonance imaging (right atrium: **light blue**; right ventricle: **dark blue**; left atrium: **yellow**; left ventricle: **red**; left ventricular myocardium: **green**). Motion analysis was used to derive strain and strain rates (radial strain in diastole and systole shown). Regional analysis of left ventricular (LV) wall thickness was performed by using 3-dimensional modeling. Mean wall thickness for 21,322 UK Biobank participants is mapped onto the LV surface; the right ventricle is shown as a mesh. **(B)** Histogram of maximum LV wall thickness according to sex.

Participants in the imaging substudy were randomly invited, and the response rates with exclusion criteria have been previously reported (11). Each underwent CMR at 1.5-T (12). Segmentation of the cine images was performed by using a deep learning neural network algorithm developed in-house and optimized on the UKBB cohort. The performance of image annotation using this algorithm is equivalent to a consensus of expert human readers and achieves subpixel accuracy for cardiac segmentation (13). Myocardial wall thickness (WT) was measured along radial line segments connecting the endocardial and epicardial surfaces perpendicular to the myocardial center-line and excluding trabeculae (Figure 1), an approach that also exceeds the reliability of human experts (14). Chamber volumes and mass were calculated from the segmentations according to standard post-processing guidelines (15). Myocardial strain analysis was performed by using nonrigid free-form deformation image registration (16,17). Trabecular traits were quantified by using fractal dimension analysis in which a higher value indicates more complex trabeculation (18). Parametric 3-dimensional analysis of the geometry of the left ventricle was performed to map regional patterns of remodeling and quantify the association with genetic and environmental predictors (16,19, 20, 21). Further details on phenotyping are given in the Supplemental Appendix.

### Sequencing and variant categorization pipeline

UKBB participants underwent WES as previously described (22). They were divided into 3 genetic strata. Individuals were classified as genotype negative (SARC-NEG) if they had no rare protein-altering genetic variation (minor allele frequency <0.001 in the UKBB and the Genome Aggregation Database) (23) in any of 25 genes that may cause or mimic HCM. These 25 genes represent an inclusive list of 8 sarcomere-encoding genes with definitive evidence of an association with HCM, 3 moderate-evidence sarcomere-encoding genes, and 14 genes associated with syndromic phenotypes that can include LVH (3). This SARC-NEG group was compared with individuals with rare variants in 8 sarcomere-encoding genes definitively associated with HCM (*MYBPC3, MYH7, MYL2, MYL3, TNNI3, TNNT2, TPM1,* and *ACTC1*). Analysis was restricted to robustly disease-associated variant classes for each gene and to variants sufficiently rare to cause penetrant disease (filtering allele frequency <0.00004) (24).

Variants were classified as pathogenic/likely pathogenic (SARC-HCM-P/LP) if reported as P/LP for HCM in ClinVar and confirmed by manual review (n = 81), or if annotated as P/LP according to ACMG criteria, using the semi-automated CardioClassifier decision support tool (24) (n = 19); the curation is depicted in Supplemental Figure 1. Other variants that were consistent with known disease mechanisms and sufficiently rare, but not recorded in ClinVar and without other computationally available data to robustly classify as P/LP, were defined as indeterminate sarcomeric variants (SARC-IND) (25,26). Despite being curated for HCM, given that 5 of the 8 genes (*MYH7, ACTC1, TNNT2, TNNI3,* and *TPM1*) that have definitive evidence for HCM have also been implicated in DCM (27), it is possible that some SARC-IND variants have the potential to cause DCM. The SARC-IND strata differs from those classically termed variants of unknown significance as it likely contains additional variants that would be reported as P/LP if subject to full manual curation, which is not feasible in >5,000 individuals (further subgroup investigations are presented in the Supplemental Appendix). Individuals harboring variants that did not fit into these 3 categories were removed from analyses, including those with rare variants in genes associated with HCM genocopies, those with intermediate-frequency variants (0.00004 < allele frequency <0.001), those with variant classes not robustly established as disease-causing (eg, truncating variants in *MYH7*), and those identified as P/LP for DCM (n = 7) (3). Details on the variant curation pipelines are presented in the Supplemental Appendix.

### Outcome measures

The effect of genotype strata on clinical outcomes was assessed by using lifetime risk. The UKBB reports the date of first occurrence of a diagnosis, identified from self-reporting, primary care, hospital in-patient, and death register records. This permitted the identification of events preceding recruitment to the UKBB. The primary clinical outcome was a composite of all-cause mortality or major adverse cardiovascular events (MACE) defined as a diagnostic code for heart failure (including cardiomyopathy), arrhythmia, stroke, or cardiac arrest events. Secondary clinical outcomes were the individual components of the primary clinical outcome. A full list of endpoint definitions and data fields used from the UKBB database is presented in the Supplemental Appendix and Supplemental Table 2.

### Statistical analysis

Statistical analysis was performed with R version 3.6.0 (R Foundation for Statistical Computing) and RStudio Server version 1.043, unless otherwise stated. Variables are expressed as percentages if categorical, mean ± SD if continuous and normal, and median (interquartile range) if continuous and non-normal. Baseline anthropometric data were compared by using Kruskal-Wallis tests and, if differences were identified, a Wilcoxon test was used for pairwise comparisons with Benjamini-Hochberg adjustment for multiple testing. Imaging parameters in 2 or more groups were compared by using analysis of covariance, adjusted for relevant clinical covariates. When differences were significant, a Tukey post hoc test was applied for pairwise multiple comparisons. Three-dimensional phenotypic regression modeling applied threshold-free cluster enhancement and permutation testing to derive the *P* values associated with each regression coefficient following adjustment to control the false discovery rate, as previously described (16,20).

Clinical outcomes were analyzed in participants with WES stratified according to genotype categories. Cox proportional hazards were calculated for lifetime risk of clinical events. For the primary outcome, competing risk analysis was performed by using the cause-specific survival method (28). Secondary analysis of incident clinical events from recruitment excluded individuals with events preceding enrollment and was performed by using Cox proportional hazards adjusted for age at recruitment. Time-to-event was censored at first event for each outcome, death, or last recorded follow-up. The relationship between genotype or CMR phenotypes and outcomes was assessed with multivariable Cox proportional hazards models and robust SE estimates. Sex was included as a covariate in all models. Hazard proportionality assumption was tested by using Schoenfeld residuals. Sex was found to be in violation of these assumptions, and therefore a sex-stratified analysis was conducted with interaction coefficients. Outcomes are reported as hazard ratios (HRs) with 95% confidence intervals (CIs) and presented graphically as cumulative hazards and Cox proportional hazards curves.

## Results

### Participants

**Figure 2:**
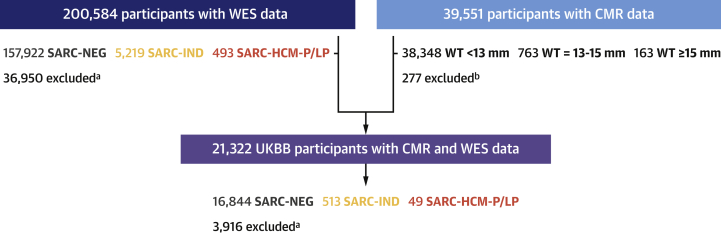
Flowchart of UKBB Participants We included 200,548 participants with whole exome sequencing (WES) in the UK Biobank (UKBB) and stratified them according to variant pathogenicity for outcome analysis. Machine learning was also used to characterize left ventricular traits in 39,551 participants, of whom 21,322 also had sequencing. ^a^Individuals excluded if carriers of: 1) rare variants in genes associated with HCM genocopies or LV phenotype; 2) intermediate frequency variants (0.00004 < AF < 0.001); 3) variant classes not robustly established as disease causing. ^b^CMRs excluded from WT measure due to nondiagnostic imaging, incomplete sequences, and other technical reasons. AF = allele frequency; CMR = cardiac magnetic resonance imaging; HCM = hypertrophic cardiomyopathy; SARC-HCM-P/LP = pathogenic or likely pathogenic variants for hypertrophic cardiomyopathy in sarcomere-encoding genes; SARC-IND = indeterminate variants in hypertrophic cardiomyopathy–associated sarcomere-encoding genes (rare variants that do not meet criteria for pathogenic/likely pathogenic annotation); SARC-NEG = genotype negative; WT = wall thickness.

We analyzed WES data from 200,584 participants and CMR from 39,551 subjects. A total of 21,322 had both CMR and WES data available (Figure 2, Table 1).

**Table 1:** Subject Characteristics and CMR-Derived Cardiac Measurements According to Genotype

	SARC-NEG (n = 157,922)	SARC-IND (n = 5,219)	SARC-HCM-P/LP (n = 493)	SARC-NEG vs SARC-HCM-P/LP	SARC-NEG vs SARC-IND	SARC-IND vs SARC-HCM-P/LP
Female	86,710 (54.9)	2,866 (54.9)	276 (56.0)			
Age, y	56.5 ± 8.1	56.3 ± 8.2	56.2 ± 8.1			
White	150,403 (95.2)	4,756 (91.1)	461 (93.5)	0.11	**<0.001**	0.11
SBP, mm Hg	139.7 ± 19.6	139.5 ± 19.8	139.2 ± 20.2			
DBP, mm Hg	82.2 ± 10.7	82.2 ± 10.5	81.4 ± 10.7			
BSA, m^2^	1.9 ± 0.22	1.9 ± 0.22	1.9 ± 0.22			
eGFR, mL/min/1.73 m^2^	90.7 ± 13.4	91.1 ± 13.3	90.1 ± 13.3			
Hypercholesterolemia	29,137 (18.5)	950 (18.2)	94 (19.1)			
Hypertension	52,356 (33.2)	1,723 (33.0)	168 (34.1)			
Diabetes	8,429 (5.3)	309 (5.9)	25 (5.1)			
Aortic valve disease	1,567 (1.0)	37 (0.7)	8 (1.6)			
Known HCM	109 (0.07)	9 (0.17)	18 (3.65)	**<0.001**	**0.006**	**<0.001**
Known DCM	265 (0.17)	9 (0.17)	1 (0.2)			
On medication for cholesterol, blood pressure, diabetes	18,537 (11.7)	599 (11.5)	64 (13.0)			

	SARC-NEG (n = 16,844)	SARC-IND (n = 513)	SARC-HCM-P/LP (n = 49)			
LVEDV, mL	147.9 ± 33.5	150.1 ± 34.6	139.4 ± 30.3	0.18	**0.005**	**0.02**
LVESV, mL	60.4 ± 19	61.4 ± 19.7	54.7 ± 15.9	0.07	**0.04**	**0.01**
LVEF, %	59.5 ± 6.1	59.5 ± 6.1	61.1 ± 5.9			
LVM, g	86 ± 22.1	87.1 ± 23.6	86.2 ± 26.6	0.43	**0.001**	0.99
LV maximum WT, mm	9.4 ± 1.6	9.5 ± 1.7	10.9 ± 2.7	**<0.001**	**0.008**	**<0.001**
LV concentricity, g/mL	0.58 ± 0.09	0.58 ± 0.09	0.62 ± 0.12	**0.001**	0.95	**0.002**
LV global longitudinal strain, %	–18.5 ± 2.8	–18.4 ± 2.8	–19 ± 2.5			
LV global radial strain, %	45 ± 8.3	44.8 ± 8.1	46.7 ± 9.3			
LV global circumferential strain, %	–22.3 ± 3.4	–22.2 ± 3.3	–22.8 ± 3.4			
LV longitudinal PDSR	1.7 ± 0.6	1.6 ± 0.6	1.7 ± 0.6			
LV radial PDSR	–5.7 ± 2.0	–5.7 ± 1.9	–5.9 ± 2.0			
LAEDV, mL	72.7 ± 23.1	73.6 ± 27.8	81.8 ± 26.8	**0.004**	0.29	**0.02**
LAESV, mL	29.2 ± 15.3	30.2 ± 20.8	34.4 ± 15.4	**0.03**	0.18	0.12
LAEF, %	61.3 ± 9.2	60.9 ± 9.8	59.2 ± 8.2			
RVEDV, mL	156.6 ± 37.4	157.7 ± 37.6	138.6 ± 32.2	**<0.001**	0.08	**<0.001**
RVESV, mL	67.6 ± 21.3	68.1 ± 21	55 ± 15.3	**<0.001**	0.26	**<0.001**
RVEF, %	57.3 ± 6.2	57.2 ± 5.9	60.6 ± 5.4	**<0.001**	0.99	**<0.001**
RAEDV, mL	86 ± 27.7	89.1 ± 32.9	80.6 ± 27.9	0.41	**0.007**	0.08
RAESV, mL	46.1 ± 19.1	47.9 ± 24.4	42.2 ± 19.4	0.33	**0.03**	0.08
RAEF, %	47.1 ± 9.4	47.5 ± 9.8	48.9 ± 9.9			
Mean apical FD	1.14 ± 0.04	1.14 ± 0.04	1.16 ± 0.05	**0.006**	0.88	**0.02**
Mean basal FD	1.19 ± 0.03	1.19 ± 0.03	1.2 ± 0.04			
Mean global FD	1.17 ± 0.03	1.17 ± 0.03	1.18 ± 0.03	**0.002**	0.96	**0.003**

Values are n (%) or mean ± SD. Table includes data for genotype-negative individuals (SARC-NEG), individuals with pathogenic or likely pathogenic sarcomeric variants (SARC-HCM-P/LP), or those with other rare indeterminate sarcomeric variants (SARC-IND). **Bold***P* values are statistically significant. Kruskal-Wallis tests were conducted for each variable to determine whether differences in participants’ characteristics between genotype group were significant; if so, a Wilcoxon test was used to perform pairwise comparisons between groups, with Benjamini-Hochberg adjustment for multiple testing, and those adjusted *P* values are shown. For the cardiac magnetic resonance imaging (CMR)-derived cardiac parameters, analysis was adjusted for age, sex, race, systolic blood pressure (SBP), and body surface area (BSA) using an analysis of covariance; when differences between genotype groups were significant, a Tukey post hoc test was applied for pairwise multiple comparisons, and those *P* values are shown.

concentricity = (left ventricular mass/left ventricular end-diastolic volume); DBP = diastolic blood pressure; DCM = dilated cardiomyopathy; eGFR = estimated glomerular filtration rate; FD = fractal dimension; HCM = hypertrophic cardiomyopathy; LAEDV = left atrial end-diastolic volume; LAEF = left atrial ejection fraction; LAESV = left atrial end-systolic volume; LV = left ventricular; LVEDV = left ventricular end-diastolic volume; LVEF = left ventricular ejection fraction; LVESV = left ventricular end-systolic volume; LVM = left ventricular mass; PDSR = peak diastolic strain rate; RAEF = right atrial ejection fraction; RVEDV = right ventricular end-diastolic volume; RVESV = right ventricular end-systolic volume; WT = wall thickness.

### Prevalence of rare sarcomeric variants

There were 157,922 SARC-NEG subjects, 493 (0.25%) heterozygotes for 100 SARC-HCM-P/LP variants, including 16 compound heterozygotes of a SARC-HCM-P/LP variant and a SARC-IND variant, and 5,219 (2.6%) heterozygotes with SARC-IND variants, including 112 compound heterozygotes where neither variant met criteria to be classified as SARC-HCM-P/LP (Supplemental Table 3).

### Left ventricular wall thickness in the general population

Of the 39,274 subjects who underwent CMR analysis, 763 (1.9% [1 in 51]) had mild hypertrophy (WT 13-15 mm) and 163 (0.4% [1 in 241]) had at least moderate hypertrophy (WT ≥15 mm). Participants with WT ≥13 mm were older (66.3 ± 7.4 years vs 63.6 ± 7.6 years; *P* < 0.001), more often male (88.4% vs 46.9%; *P* < 0.001), and with higher systolic blood pressure (149.5.2 ± 18.8 mm Hg vs 138 ± 18.2 mm Hg; *P* < 0.001) and body surface area (2.07 ± 0.21 m^2^ vs 1.86 ± 0.21 m^2^; *P* < 0.001). There were no differences in terms of race. The prevalence of phenotypic HCM, defined as WT ≥15 mm in the absence of hypertension and valve disease, was 0.19% (n = 76 [1 in 517]).

### Penetrance and expressivity of rare sarcomeric variants

**Figure 3:**
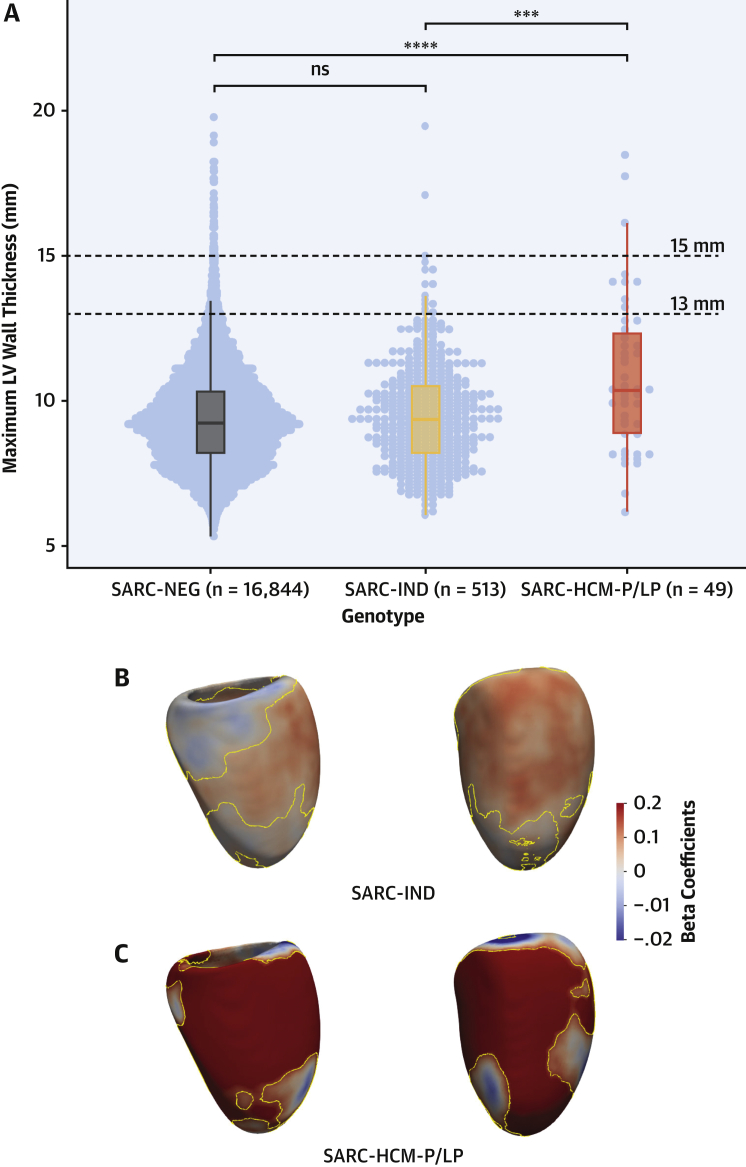
Relationship Between Rare Variants in HCM-Associated Genes and WT **(A)** Dot and boxplots of maximum wall thickness according to genotype. **(B and C)** 3-dimensional modeling of LV geometry with standardized beta-coefficients showing the strength of association between genotype and regional WT. Contour lines indicate significant regions (*P* < 0.05) after correction for multiple testing. LV projections are septal **(left)** and anterior **(right)**. ∗∗∗*P* ≤ 0.001; ∗∗∗∗*P* ≤ 0.0001. ns = not significant; other abbreviations as in Figures 1 and 2.

Analyses were restricted to UKBB participants with both CMR and WES (n = 21,322). In this subset, 16,844 were denoted as SARC-NEG, 513 as SARC-IND, and 49 as SARC-HCM-P/LP. CMR-derived cardiac measurements by genotype are summarized in Table 1. Wall thickness was greater in SARC-HCM-P/LP versus SARC-NEG (Figure 3) and marginally greater in SARC-IND after adjustment for relevant clinical variables. Compared with SARC-NEG, those harboring SARC-HCM-P/LP variants showed evidence of concentric remodeling, smaller right ventricular volumes, higher left atrial volume, and increased trabeculation. Independent, 3-dimensional analysis of cardiac geometry showed that SARC-IND variants were positively associated with an asymmetric increase in WT (n = 508; mean β = 0.08; significant area = 70.1%), predominantly across the mid-basal anterior septum in continuity with the anterior wall. There was a much stronger positive association between SARC-HCM-P/LP variants and increased WT (n = 48; mean β = 0.31; significant area = 47%) in an asymmetric pattern involving most of the septum, anterior wall, and apex.

In the 49 subjects harboring SARC-HCM-P/LP variants, LVH ≥13 mm was present in 9, corresponding to a penetrance of 18.4% (95% CI: 9%-32%) for this phenotype. In 3 of these individuals, LVH was ≥15 mm (6.1%; 95% CI: 1.3%-16.8%). Of the 513 with SARC-IND variants, 15 individuals (2.9%; 95% CI: 1.6%-4.8%) had WT ≥13 mm. In 390 of these 562 individuals with a SARC-IND or SARC-HCM-P/LP variant, there was no concomitant hypertension or valve disease (69.4%). Among these 390 individuals, the penetrance of LVH ≥13 mm was 10.8% for SARC-HCM-P/LP (4 of 37) and 0.57% for SARC-IND (2 of 353). Details of variants found in these 6 individuals with otherwise unexplained LVH are shown in Supplemental Table 4. Among the 173 individuals with a rare sarcomere-encoding variant and hypertension or valve disease, the penetrance of LVH was 41.7% for SARC-HCM-P/LP (5 of 12) and 8.1% for SARC-IND (13 of 161). Only 1 individual of the 39 with a wall thickness ≥15 mm (2.6%), not accounted for by hypertension or valve disease, harbored a SARC-HCM-P/LP variant. There was no difference in the prevalence of left ventricular ejection fraction <50% (*P* = 0.12) between subjects harboring SARC-HCM-P/LP variants (1 of 49 [2%]; 95% CI: 0.0%-10.9%), subjects with a SARC-IND variant (29 of 513 [5.7%]; 95% CI: 3.9%-8.0%), and those identified as SARC-NEG (888 of 16,844 [5.3%]; 95% CI: 4.9%-5.6%). The prevalence of left ventricular dilatation (defined by using the UKBB-derived normal ranges of left ventricular end-diastolic volume [abnormal high >232 mL in male subjects and >175 mL in female subjects]) (29) was also not different in SARC-NEG individuals (530 of 16,844 [3.15%]; 95% CI: 2.9%-3.4%) and those with SARC-IND (16 of 513 [3.12%]; 95% CI: 1.8%-5.0%) or SARC-HCM-P/LP (0 of 49 [0%]; 95% CI: 0.0%-7.3%) variants.

### Clinical outcomes associated with rare sarcomeric variants

Clinical outcomes for 163,634 participants were analyzed. Median age at recruitment was 58 years (interquartile range: 50-63 years), and participants were followed up for a median of 10.8 years (interquartile range: 9.9-11.6 years) with a total of 19,504 primary clinical events reported. Among SARC-NEG (n = 157,922), SARC-IND (n = 5,219), and SARC-HCM-P/LP (n = 493) individuals, there were 18,793 (11.9%), 616 (11.8%), and 95 (19.3%) primary clinical outcome events (all-cause mortality or MACE), and by age 70 years there were 14,168 (cumulative incidence: 12%; 95% CI: 12%-12%), 461 (cumulative incidence: 12%; 95% CI: 11%-13%), and 80 events (cumulative incidence: 21%; 95% CI: 16%-25%), respectively.

**Figure 4:**
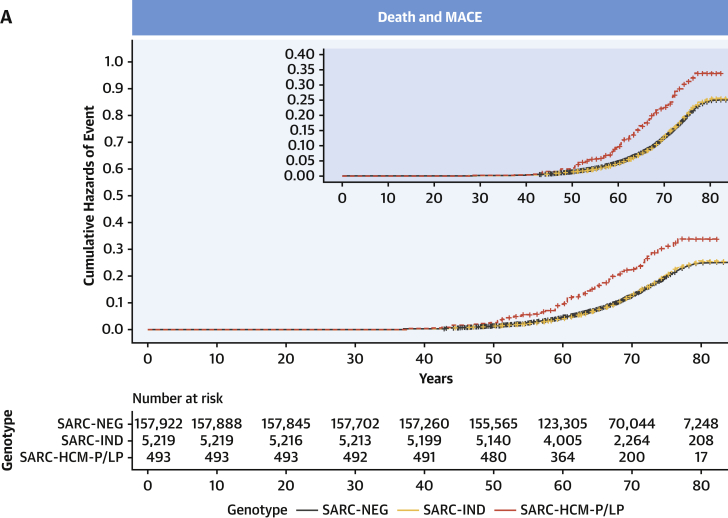

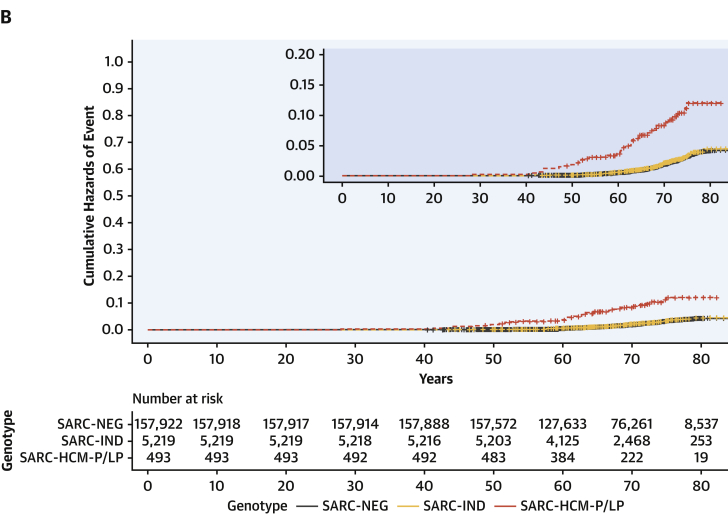

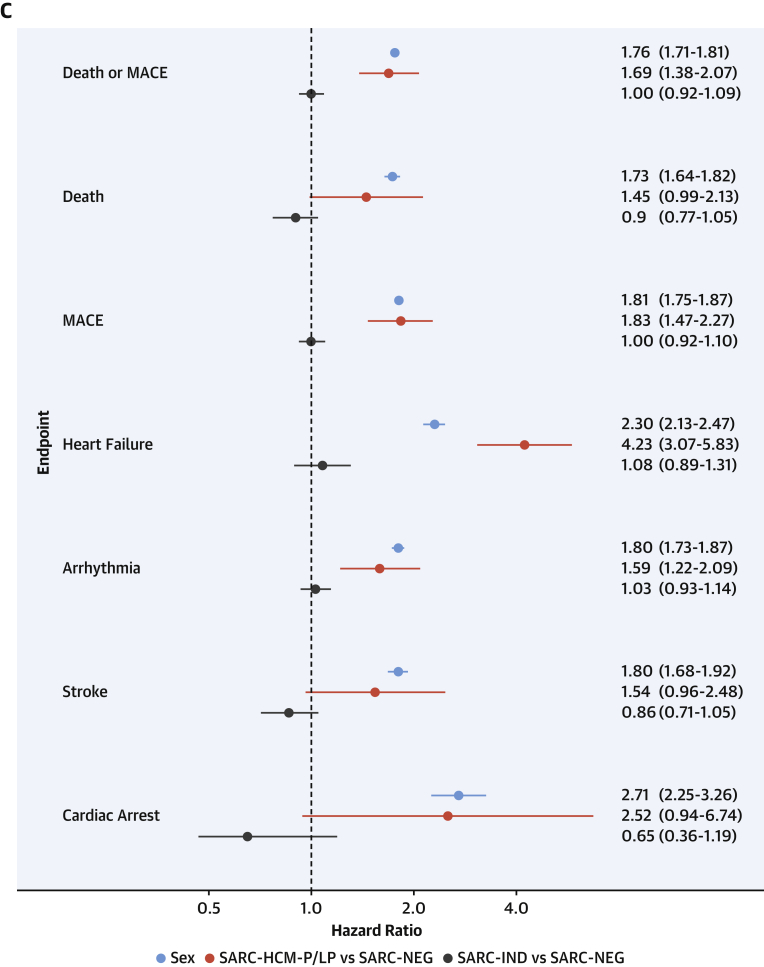
Outcomes Stratified According to Variant Pathogenicity Cumulative hazard curves with zoomed plots for lifetime risk of: **(A)** death and major adverse cardiovascular events (MACE), consisting of heart failure, arrhythmia, stroke, and cardiac arrest events, or **(B)** heart failure, stratified according to genotype, consisting of SARC-NEG, SARC-IND, or SARC-HCM-P/LP. **(C)** Forest plot of comparative lifetime risk of clinical endpoints (Cox proportional hazards models adjusted for sex) according to genotype. Sex refers to male subjects compared with female subjects. Abbreviations as in Figure 2.

Examining lifetime risks, SARC-HCM-P/LP variants were associated with an increased risk of death or MACE (HR: 1.69; 95% CI: 1.38-2.07; *P* < 0.001), heart failure (HR: 4.23; 95% CI: 3.07-5.83; *P* < 0.001), and arrhythmia (HR: 1.59; 95% CI: 1.22-2.09; *P* < 0.001) (Figure 4, Supplemental Table 5). There was no difference in risk in any of the primary or secondary clinical outcomes when comparing SARC-IND and SARC-NEG. Sex was independently associated with all clinical outcomes, with men having an increased risk. Although male subjects have a higher overall risk of adverse outcomes (HR: 1.76; 95% CI: 1.71-1.81; *P* < 0.001), the incremental genetic risk from SARC-HCM-P/LP mutations compared with SARC-NEG is greater in female subjects (HR for female subjects: 2.24 [95% CI: 1.71-2.94; *P* < 0.001]; HR for male subjects: 1.31 [95% CI: 0.97-1.76; *P* = 0.08]; HR for sex∗SARC-HCM-P/LP interaction: 1.72 [95% CI: 1.15-2.58; *P* = 0.009]) (Supplemental Table 6, Supplemental Figure 2). Sensitivity analyses of lifetime risk of death or MACE, excluding participants with any cardiomyopathy and excluding cardiomyopathy events as an outcome, yielded concordant results to the primary analysis comparing SARC-HCM-P/LP with SARC-NEG (excluding participants with cardiomyopathy HR: 1.29 [95% CI: 1.02-1.63; *P* = 0.03]; excluding cardiomyopathy diagnosis from the HF composite outcome HR: 1.60 [95% CI: 1.25-2.04; *P* < 0.001]) (Supplemental Table 7), confirming that previous findings were not solely driven by individuals with known disease, nor only by a diagnostic label of cardiomyopathy as an endpoint. Examining incident risks, primary clinical events (HR: 1.57; 95% CI: 1.2-2.04; *P* < 0.001), including heart failure (HR: 3.15; 95% CI: 2.03-4.89; *P* < 0.001), were increased in SARC-HCM-P/LP but not in SARC-IND (Supplemental Table 8, Supplemental Figure 3).

In the 39,551 individuals who underwent CMR, the number of primary clinical events was 2,568 (6.7%), 112 (14.7%), and 34 (20.9%) in the WT <13 mm, 13-15 mm, and ≥15 mm subgroups, respectively. Increased WT was associated with an increased risk of death or MACE (per millimeter increase HR: 1.15; 95% CI: 1.11-1.20; *P* < 0.001) and each individual endpoint (Supplemental Table 9, Supplemental Figure 4), with similar findings in the imaging and WES subgroup (n = 17,447) (Supplemental Table 10, Supplemental Figure 5). Increased trabecular complexity, as measured by fractal dimension analysis, was associated with adverse clinical outcomes (HR per 0.1 unit increase in mean global fractal dimension: 1.04; 95% CI: 1.01-1.08; *P* = 0.01) after adjustment for systolic blood pressure and WT (Supplemental Table 11, Supplemental Figure 6).

Among 17,406 individuals in the 3 genotype strata with imaging and WES data, there were 1,177 (7.0%), 44 (8.3%), and 87 (16.0%) primary clinical events in SARC-NEG, SARC-IND, and SARC-HCM-P/LP, respectively. The risk of heart failure was higher with SARC-HCM-P/LP variants despite adjustment for WT (HR: 6.74; 95% CI: 2.43-18.7; *P* < 0.001) (Supplemental Table 12) and also when accounting for WT, left atrial volume, left ventricular ejection fraction, and left ventricular end-diastolic volume (HR: 8.05; 95% CI: 2.46-26.3; *P* < 0.001) (Supplemental Tables 13 and 14).

## Discussion

In this study of >200,000 adults, we found the prevalence of rare variants in HCM-associated sarcomere-encoding genes to be 2.9%, with the prevalence of SARC-HCM-P/LP variants conservatively measured at 0.25%, both less frequent than reported in smaller cohorts (30), prior to contemporary variant classification guidelines (25) and a consensus emerging on genes with definitive evidence for disease causation in HCM (3). By examining lifetime risks alongside precision cardiac phenotyping, we provide a critical evaluation of large-scale genomic screening for sarcomeric variants outside of familial disease (Central Illustration).

**Central Illustration:**
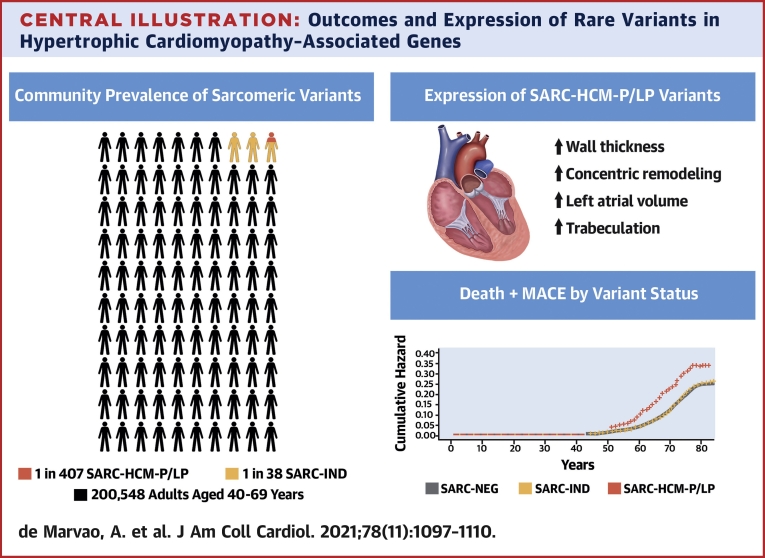
Outcomes and Expression of Rare Variants in Hypertrophic Cardiomyopathy–Associated Genes In 200,000 adults, the prevalence of variants pathogenic or likely pathogenic for hypertrophic cardiomyopathy (SARC-HCM-P/LP) was 1 in 407, whereas the aggregate prevalence of indeterminate sarcomeric variants was 1 in 38. The SARC-HCM-P/LP variants were associated with increased risk of death and major adverse cardiovascular events. We found associations with hypertrophic cardiomyopathy–like imaging phenotypes although the prevalence of overt cardiomyopathy was low. SARC-IND = indeterminate variants in hypertrophic cardiomyopathy–associated sarcomere-encoding genes (rare variants that do not meet criteria for pathogenic/likely pathogenic annotation); SARC-NEG = genotype negative.

We found that SARC-HCM-P/LP variants were associated with an increased lifetime risk of adverse cardiovascular events, predominantly due to heart failure and arrhythmia, which was partially independent of left ventricular wall thickness and not restricted to individuals with cardiomyopathy or reaching cardiomyopathy endpoints. The fact that heart failure risk is only partially explained by observable phenotypic expressivity is consistent with the profibrotic state that precedes the development of LVH in those with HCM variants (31). This finding highlights the potential of genomic testing for sarcomeric variants to modify cardiovascular risk assessment even in the absence of image phenotyping. Such variants are also associated with greater risk in HCM patient cohorts, with adverse outcomes reported to be twice as likely in patients with HCM harboring SARC-HCM-P/LP variants (32). SARC-HCM-P/LP variants were associated with a greater increase in lifetime risk of death and MACE in female subjects than in male subjects. This supports findings in patients with HCM, in whom, despite apparent reduced disease penetrance (33), women have lower survival regardless of genotype (34,35). In contrast, SARC-IND variants (which include a mixture of variants with potential to cause HCM, DCM, or have little impact on cardiomyopathy risk) express a minimal phenotype in aggregate and have a generally benign clinical course. Recent study shows that the same genetic pathways may lead to distinct disorders through opposing genetic effects in the general population (36).

Adults harboring SARC-HCM-P/LP variants had an attenuated HCM-related phenotype characterized by increased wall thickness in an asymmetric pattern involving the anteroseptum and apex, concentric ventricular remodeling, increased left atrial volume, and greater trabeculation. In subjects harboring SARC-HCM-P/LP variants, hypertrophy (≥13 mm) was present in 18.4%, with 3 individuals presenting with hypertrophy ≥15 mm (6.1%). Penetrance estimates from family studies vary, but a recent study of relatives of HCM probands observed an HCM phenotype in 37% of genotype-positive relatives at first screening (37). Although UKBB participants have a higher degree of kinship than would be expected in a random sample (38), these results suggest that the penetrance of familial HCM is additionally driven by other genetic or environmental disease–modifying characteristics, shared within families but that overlap less commonly in the community. Conversely, only 2.6% of individuals with unexplained LVH (WT ≥15 mm) harbored SARC-HCM-P/LP variants. This contrasts with the diagnostic yield in HCM cohorts, in which even patients without family history have a comparatively high 30% yield of sarcomere variants (39), suggesting that patients enrolled into cohorts and/or referred for diagnostic sequencing represent a skewed subset, likely enriched by those with clinical risk factors (40,41). Although the natural history of disease and family pedigrees are not known for UKBB participants with unexplained LVH, it is plausible that multifactorial sarcomere-negative hypertrophy is the predominant etiology in the community (42,43). Nonetheless, increasing WT was associated with higher risk of death and MACE (and each individual component), which persisted despite adjustment for sex, sarcomeric genotype, and systolic blood pressure. These findings illustrate the additive role of clinical and genetic assessment in risk stratifying patients with unexplained LVH and managing the appropriate screening of relatives (2).

### Study limitations

UKBB is a large-cross sectional study that is subject to selection bias, excluding younger and potentially more severe cases (44); however, risk factor associations seem to be broadly generalizable (45). The population is predominantly European, and further work is required to explore traits and outcomes in people of diverse ancestries. Although we included outcome data before enrollment in the UKBB, we do not know the natural history of disease in the community or what factors may influence conversion to penetrant disease; in addition, co-segregation data were not available. Although there is genetic overlap between HCM and DCM, we found that DCM traits do not drive clinical outcomes in individuals with SARC-IND or SARC-HCM-P/LP variants.

## Conclusions

We found that SARC-HCM-P/LP variants are present in 1 in 407 adults. Although the presence of SARC-HCM-P/LP variants in individuals in the community was rarely associated with the degree of unexplained hypertrophy required for a diagnosis of HCM, they are associated with an attenuated phenotype and an increased risk of adverse cardiovascular events even in the absence of cardiomyopathy. SARC-HCM-P/LP variants are likely to be harbored by >18 million people worldwide, and an improved understanding of their clinical significance is crucially important, especially in light of recommendations to actively screen for such variants as secondary findings during clinical sequencing (46). Although penetrance for overt HCM is modest and absolute event rates are low, identification of these variants may enhance risk stratification beyond familial disease, even when cardiomyopathy is not manifest.

Perspectives **COMPETENCY IN MEDICAL KNOWLEDGE:** Most individuals with pathogenic sarcomeric variants do not have overt HCM, but a subclinical phenotype is associated with an increased risk of adverse cardiovascular events.

**TRANSLATIONAL OUTLOOK:** Further genomic analyses are needed to characterize the specific risks levels and types of complications associated with various inherited forms of HCM.

## Funding Support and Author Disclosures

This study was supported by the Medical Research Council, UK (MC-A658-5QEB0); the National Institute for Health Research Imperial College Biomedical Research Centre; the National Institute for Health Research Royal Brompton Cardiovascular Biomedical Research Unit; the British Heart Foundation (NH/17/1/32725, RG/19/6/34387, RE/18/4/34215); Fondation Leducq (16 CVD 03); Wellcome Trust (107469/Z/15/Z, 200990/A/16/Z); the National Heart and Lung Institute Foundation; the Royston Centre for Cardiomyopathy Research; Rosetrees and CORDA (Dr Prasad); Academy of Medical Sciences (SGL015/1006; Dr de Marvao); Mason Medical Research Trust grant (Dr de Marvao); SmartHeart EPSRC Programme Grant (EP/P001009/1; Dr Bai and Dr Rueckert); and a Rosetrees and Stoneygate Imperial College Research Fellowship (Dr Whiffin). Dr Ware has consulted for MyoKardia, Inc. and Foresite Labs. Dr Cook holds shares in Enleofen Bio Pte. Ltd. Dr O’Regan has consulted for Bayer AG. All other authors have reported that they have no relationships relevant to the contents of this paper to disclose.
